# Prevalence and associated clinical factors for overweight and obesity in young first-episode and drug-naïve Chinese patients with major depressive disorder

**DOI:** 10.3389/fpsyt.2023.1278566

**Published:** 2023-10-18

**Authors:** Jian-Jun Zhang, Xiao-Qian Wang, Qun Zeng, Na Gao, Xiang-Yang Zhang

**Affiliations:** ^1^Shanxi Key Laboratory of Chinese Medicine Encephalopathy, National International Joint Research Center for Molecular Chinese Medicine, Shanxi University of Chinese Medicine, Taiyuan, China; ^2^CAS Key Laboratory of Mental Health, Institute of Psychology, Beijing, China; ^3^College of Basic Medical Sciences, Shanxi University of Chinese Medicine, Jinzhong, China; ^4^Department of Cardiology, The Second Medical Center & National Clinical Research Center for Geriatric Diseases, Chinese People’s Liberation Army (PLA) General Hospital, Beijing, China; ^5^Department of Psychology, University of Chinese Academy of Sciences, Beijing, China

**Keywords:** overweight, obesity, major depressive disorder (MDD), young, first episode and drug naïve (FEDN) patients

## Abstract

**Background:**

Obesity and overweight are common in young patients with major depressive disorder (MDD). However, the prevalence and associated clinical factors of obesity/overweight in young first-episode and drug-naïve (FEDN) MDD patients are rarely reported in China.

**Methods:**

A cross-sectional study of 917 young patients (aged 18–35 years) with FEDN MDD was performed. Demographic and clinical data were collected. Depression, anxiety, and psychotic symptoms were assessed using the Hamilton Depression Scale (HAMD), the Hamilton Anxiety Scale (HAMA), and the Positive and Negative Syndrome Scale (PANSS) positive subscale, respectively.

**Results:**

Among the young MDD patients, the prevalence of obesity and overweight was 4.14 and 52.89%, respectively. Compared to normal-weight patients, overweight patients were older, had a greater age of onset, and had higher TSH and TG levels. Male MDD patients had a higher risk of obesity than female patients. Compared to obese patients, normal-weight and overweight patients had significantly lower HAMD scores, TC levels, and rates of TSH abnormalities. Logistic regression analysis showed that age, age of onset, and sex were independently associated with obesity, and TSH was independently associated with both obesity and overweight, in young MDD patients.

**Conclusion:**

Our findings suggest a high prevalence of overweight and obesity in young FEDN MDD patients. Several demographic and clinical variables are independently associated with overweight/obesity in these young MDD patients.

## 1. Introduction

The prevalence of major depressive disorder (MDD), especially among young adults, is increasing each year and is of growing concern. One study showed that the prevalence of MDD among young men and young women in the United States was 6.4 and 9.2%, respectively (1). Another survey of youth across the United States showed a 14.3% prevalence of depression in the last month and a 7.8% prevalence of depression for 7 and 14 days or more (2). The youth population tends to have a higher risk of developing depression (3). In addition, vegetative symptoms, such as changes in weight and appetite, low energy, and insomnia, are more common in younger patients with MDD than in adults (4). However, only 30% of young patients achieve remission in current antidepressant treatment (5–7). Therefore, a more in-depth study of the pathogenesis of MDD in young adults is needed. It has been shown that the pathogenesis of depression in youth may involve multiple aspects, including neuroendocrine and glucose/lipid metabolism-related mechanisms (8). Compared with the general population, young MDD patients tend to have higher glucose, total and low-density lipoprotein cholesterol (LDL-C), body mass index (BMI), lower high-density lipoprotein cholesterol (HDL-C), and higher rates of abdominal obesity (9).

The prevalence of overweight and obesity in young populations varies by country. A study of young Australians showed that 20.6% of women were overweight, 20.6% of men were obese, 29.3% of men were overweight, and 13.8% of men were obese (10). Another survey of young Ugandans showed that the overall prevalence of obesity and overweight was 2.3 and 10.4%, respectively (11). A study of young Chinese university students showed an overall prevalence of 9.5% for overweight and obesity, with 13.9% in males and 6.1% in females (12).

In clinical practice, the coexistence of overweight and obesity with depression is common in young adults. A study of young adults in the United States showed a correlation between overweight/obesity and depression in a younger population, but not in an older population (2). In China, fasting glucose, HbA1c, and triglycerides were found to be risk factors for depression in young obese patients (13). There are several studies of depressed patients of all ages, and the results vary across geographic regions. A German survey of depressed patients who changed medication early showed that 31% were overweight and 21% were obese (14). A Portuguese survey of depressed patients showed that 27% of patients were overweight and 46% were obese (15). A survey of outpatients with depression in the United States showed that 51.4% of patients were overweight and 20.0% were classified as obese (16). However, the geographical limitation of patients and the BMI of subjects in these studies may be influenced by antidepressants, which may lead to different results. Our team’s recent survey of depressed patients of all ages in China showed that 3.73% were obese and 56.00% were overweight (17). However, this study was an all-age study of adult depressed patients, whereas there are relatively few studies on the prevalence of obesity and overweight in the young depressed population in China, and associated clinical factors are unclear.

To date, studies on the prevalence of obesity and overweight in young depressed patients are relatively scarce. Therefore, to reduce the effect of medications and other factors on BMI (18, 19), we recruited 917 first-episode and drug naïve MDD patients aged 18–35 years in mainland China as study subjects and collected clinical data and lipid metabolism and neuroendocrine-related biomarkers. The main objectives of this study were (1) to investigate the prevalence of obesity and overweight in young MDD patients, and (2) to examine the associated clinical factors associated with obesity and overweight in young MDD patients.

## 2. Methods

### 2.1. Study design and subjects

The study was approved by the Institutional Review Board (IRB) of the First Hospital of Shanxi Medical University. All subjects signed a written consent form prior to participation in this study.

In this cross-sectional study, 917 consecutive outpatients were recruited from the First Hospital of Shanxi Medical University. Participants were recruited in accordance with the Declaration of Helsinki. Each patient was then independently evaluated by two experienced psychiatrists according to the Chinese version of the Structured Clinical Interview for DSM-IV (SCID) to confirm the diagnosis of MDD. Subjects met the following inclusion criteria: (1) 18–35 years old, Han Chinese; (2) no previous antidepressant or antipsychotic treatment; (3) first episode of MDD; (4) duration of illness less than two years; and (5) not pregnant or breastfeeding.

After a detailed medical history review, physical examination, and laboratory testing, we acquired a complete medical history and clinical/anthropometric data for all patients. Patients meeting the following criteria were excluded: (1) axis I disease other than MDD; (2) central nervous system disease, acute, unstable, or life-threatening disease (e.g., infection, cancer, and organ failure); (3) history of drug/alcohol abuse or dependence; and (4) refusal to sign a written consent form.

### 2.2. Collection of socio-demographic information

Sociodemographic information was collected using a standard questionnaire that included gender, age, age of onset, duration of illness, marital status, education level, and suicide attempts. The study questionnaire was administered by trained research staff. BMI was calculated as weight (kg) divided by height (m) squared and patients were classified as normal weight (BMI < 24 Kg/m2), overweight (24 Kg/m2 ≤ BMI < 28 Kg/m2) or obese (BMI ≥ 28 Kg/m2) (20).

### 2.3. Clinical measurements

The HAMD-17 was used for the comprehensive assessment of depressive symptoms. The scale contains 8 items on a 5-point scale from 0 (no symptoms) to 4 (very severe symptoms) and 9 items on a 3-point scale from 0 (no symptoms) to 2 (severe symptoms). Patients with a score of 24 or more were considered to have severe depressive symptoms (21).

The PANSS positive subscale was used to assess the severity of psychotic symptoms in patients with MDD. Each item was scored from 1–7. Patients with a total score > 15 were considered to have psychotic symptoms (22).

The 14-item Hamilton Anxiety Rating Scale (HAMA) was used to quantify the severity of anxiety symptoms. Patients with a total score > 29 were considered to have severe anxiety symptoms.

The Clinical Global Impression of Severity Scale (CGI-S) was used to measure clinicians’ impression of the current severity of illness, with scores ranging from 1 (normal) to 7 (most severe) (23).

Prior to the investigation, two qualified psychiatrists were trained in the use of these rating scales. After repeated assessments, interobserver correlation coefficients for HAMD and PANSS scores exceeded 0.8, respectively.

### 2.4. Blood biomarker measurements

Blood samples were collected in the morning after an overnight fast and immediately sent to the hospital’s laboratory center for measurement on the same day. In this study, blood lipids and thyroid function were measured, including HDL-C, LDL-C, triglycerides (TG), total cholesterol (TC), free triiodothyronine (FT3), free thyroxine (FT4), and thyroid stimulating hormone (TSH). Serum TSH, FT3, and FT4 were performed by chemiluminescent immunoassay. The normal range of TSH was 0.27–4.20 mIU/L, FT3 was 3.10–6.8 pmol/L and FT4 was 10–23 pmol/L. The lipid profile was measured by enzyme-labeled colorimetric assay.

### 2.5. Statistical analysis

First, the normality of all variables was tested by the Shapiro–Wilk test, and the homogeneity of variance was determined by the Levene test. Categorical variables were expressed as frequencies and percentages, and the X^2^ test was used for comparison of categorical variables. All normally distributed continuous variables were expressed as mean ± SD, and non-normally distributed variables were expressed as median (quartiles). Comparisons of continuous variables conforming to the normal distribution were performed using analysis of variance (ANOVA), and non-normally distributed variables were tested using the Kruskal-Wallis rank sum test.

For *post hoc* analyses, paired comparisons between the three groups (normal weight, overweight, and obese) were performed using the Nemenyi test. To further explore the effect of each clinical factor on BMI, a multivariate unordered logistic regression model was constructed to clarify the effect of each factor on BMI levels (normal weight, overweight, and obesity). BMI was considered as the dependent variable.

All statistical analyses in this study were performed using the SPSS 20.0 platform, and all *p*-values were calculated using two tails, with significance set at *p* < 0.05.

## 3. Results

### 3.1. Prevalence and demographic and clinical variables of overweight or obesity in young MDD patients

As shown in Table 1, the following variables did not follow a normal distribution: age at onset, age, A-TG, A-TPO, FT3, FT4, HDL-C, LDL-C, TC, TG, HAMA, and HAMD (all *p* < 0.05). Among 917 young MDD patients (351 men and 566 women), the mean age of onset was 24 years. The mean duration of the disease was 4 months. The mean total scores of HAMD and HAMA were 30 and 21, respectively.

**Table 1:** Characteristics and prevalence of overweight/obesity of young patients with MDD.

	Total	Normal-weight (BMI < 24)	Overweight (24 ≤ BMI < 28)	Obesity (BMI ≥ 28)	*p* value
Young MDD Patients (%)	917	394 (42.97%)	485 (52.89%)	38 (4.14%)	0.249
Sex, male, *N* (%)	351 (38.3%)	149 (37.8%)	179 (36.9%)	23 (60.5%)	0.015^*^
Age (years)	24 (19.50, 29)	23 (19, 28)	25 (20, 30)	26 (20, 29.25)	0.010^*^
Age of onset (years)	24 (19, 29)	23 (19, 28)	25 (20, 30)	26 (19, 29.25)	0.012^*^
Duration (months)	4 (3, 6)	4 (2.5, 6)	4 (3, 7)	5 (2.875, 9.00)	0.216
					
Education, *N* (%)					0.357
Junior high school	57 (6.2%)	29 (7.4%)	24 (4.9%)	4 (10.5%)	
Senior high school	439 (47.9%)	182 (46.2%)	242 (49.9%)	15 (39.5%)	
College	345 (37.6%)	145 (36.8%)	185 (38.1%)	15 (39.5%)	
Postgraduate	76 (8.3%)	38 (9.6%)	34 (7.6%)	4 (10.5%)	
Marriage					0.356
Unmarried	481 (52.5%)	217 (55.1%)	246 (50.7%)	18 (47.4%)	
Married	436 (47.5%)	177 (44.9%)	239 (49.3%)	20 (52.6%)	
CGI					0.016^*^
5	300 (32.7%)	127 (32.2%)	170 (35.1%)	3 (7.9%)	
6	388 (42.3%)	170 (43.1%)	197 (40.6%)	21 (55.3%)	
7	329 (25.0%)	97 (24.6%)	118 (24.3%)	14 (36.8%)	
HAMD	30 (28, 32)	30 (28, 32)	30 (28, 32)	31.5 (30, 33)	0.015^*^
HAMA	21 (18, 23)	21 (18, 23)	20 (18, 23)	22 (19, 24)	0.146
Severe anxiety					0.235
NO	818 (89.2%)	356 (90.4%)	431 (88.9%)	31 (81.6%)	
YES	99 (10.8%)	38 (9.6%)	54 (11.1%)	7 (18.4%)	
Attempted suicide					0.013^*^
NO	738 (80.5%)	314 (79.7%)	400 (82.5%)	24 (63.2%)	
YES	179 (19.5%)	80 (20.3%)	85 (17.5%)	14 (36.8%)	
Psychotic symptoms					0.515
NO	834 (90.9%)	356 (90.4%)	445 (91.8%)	33 (86.8%)	
YES	83 (9.1%)	38 (9.6%)	40 (8.2%)	5 (13.2%)	
					
FT3	4.97 (4.39, 5.46)	4.880 (4.338, 5.430)	4.990 (4.465, 5.485)	5.180 (4.375, 5.585)	0.114
FT4	16.55 (14.51, 18.81)	16.550 (14.575, 18.670)	16.520 (14.490, 18.765)	17.570 (14.215, 21.293)	0.206
HDL-C	1.25 (1.02, 1.41)	1.250 (1.008, 1.440)	1.240 (1.025, 1.400)	1.245 (0.990, 1.430)	0.900
LDL-C	2.89 (2.30, 3.49)	2.850 (2.300, 3.500)	2.900 (2.300, 3.400)	3.360 (2.400, 3.900)	0.141
TC	5.10 (4.37, 5.92)	4.930 (4.270, 5.893)	5.130 (4.420, 5.880)	5.445 (5.116, 6.260)	0.010^*^
TG	1.91 (1.35, 2.77)	1.795 (1.278, 2.740)	2.010 (1.440, 2.780)	1.885 (1.290, 2.890)	0.043^*^
FPG	5.28 (4.89, 5.77)	5.230 (4.868, 5.770)	5.340 (4.915, 5.750)	5.560 (5.028, 5.988)	0.175
TSH					<0.000^***^
Normal (0.2–4.20mIU/L)	394 (43%)	207 (52.5%)	180 (37.1%)	7 (18.4%)	
Abnormal (>4.20mIU/L)	523 (57%)	187 (47.5%)	305 (62.9%)	319 (81.6%)	

BMI, body mass index. ^*^p < 0.05; ^***^p < 0.001.

Among these young MDD patients, the prevalence of obesity and overweight was 4.14% (38/917) and 52.89% (485/917), respectively. The chi-square analysis showed that gender was significantly different in the three groups (*p* = 0.015). The Kruskal-Wallis rank test showed that age (*p* = 0.010), age of onset (*p* = 0.012), HAMD (p = 0.015), A-TPO (*p* = 0.013), and TC (*p* = 0.010), TG (*p* = 0.043), TSH (*p* < 0.001), CGI (*p* = 0.016), and suicide attempt rate (*p* = 0.013) were significantly different among the three groups.

As shown in Table 2, *post hoc* analysis showed that overweight patients were older (*p* = 0.01), had a greater age of onset (*p* = 0.01), and had higher rates of TSH abnormalities (*p* < 0.001) and TG levels (*p* = 0.04) compared to the normal weight group. HAMD score, TC levels, and the rate of TSH abnormalities were lower in overweight patients (P_HAMD_ = 0.04, P_TC_ = 0.03, P_TSH_ = 0.02) and normal-weight patients (P_HAMD_ = 0.02, P_TC_ = 0.01, P_TSH_ < 0.001) than in the obese group.

**Table 2:** All *p* values for posthoc comparison of selected variables among normal, overweight, and obesity groups.

	Normal vs. Overweight	Normal vs. Obesity	Overweight vs. Obesity
Age(years)	0.01	0.38	0.97
Age of onset(years)	0.01	0.43	0.99
HAMD	0.62	0.02	0.04
A - TPO	0.05	0.55	0.11
TC	0.59	0.01	0.03
TG	0.04	0.95	0.79
TSH	<0.001	<0.001	0.02

### 3.2. Association between clinical parameters and risk of overweight or obesity

An ordered logistic regression model was built using three ordered categorical variables (normal weight, overweight, and obesity), and the significance indicators in Table 1 were extracted to further explore their relationship with BMI levels. Using the multivariate ordered logit model, the parallel line test was *p* = 0.000, which did not meet the requirement. Therefore, an unordered multi-classification logit regression model was used: regression models for normal-weight, obesity, and overweight were established with normal-weight as a reference.

As shown in Table 3, age and age of onset were higher in the obese group, and TSH (two categories, defined by 4.20 mol/L) was higher in both obese and overweight groups than in the normal-weight group. For each year increase in patient age, the risk of obesity was approximately 2.497 times higher than in the normal-weight group (*p* = 0.002, OR: 2.497, 95% C.I.: 1.391–4.484). For each year increase in age of onset, the risk of obesity was about 2.433 times higher than the normal-weight group (*p* = 0.003, OR: 2.433, 95% C.I.: 1.344–4.405). In young MDD patients, the risk of obesity was nearly 2.549 times higher in men than in women (*p* < 0.0001, OR: 2.549, 95% C.I.: 1.628–3.99), but there was no significant sex difference in the risk of being overweight (*p* = 0.593). Compared with normal-weight patients, the risk of obesity was 4.551 times greater in patients with TSH abnormalities than in patients with normal TSH (*p* < 0.0001, OR: 4.551, 95% C.I.: 2.419–8.563). The risk of being overweight in patients with TSH abnormalities was 2.286 times higher than in patients with normal TSH (*p* < 0.0001, OR: 2.286, 95% C.I.: 1.707–3.06).

**Table 3:** Results of multivariable disordered logit model.

		B	S.E.	Wald	df	*p* Value	OR	95%C.I.	
								Lower limit	Upper limit
Obesity	Age (years)	0.915	0.299	9.389	1	0.002	2.497	1.391	4.484
	Age of onset (years)	−1.126	0.302	8.637	1	0.003	2.433	1.344	4.405
	HAMD	−0.043	0.059	0.525	1	0.469	0.958	0.853	1.076
	ATPO	0	0.001	0	1	1	1	0.999	1.001
	TC	0.016	0.137	0.013	1	0.908	1.016	0.776	1.33
	TG	−0.066	0.126	0.278	1	0.598	0.936	0.731	1.198
	Sex								
	Male	0.936	0.229	16.745	1	0	2.549	1.628	3.99
	Female								
	TSH								
	Normal (0.2–4.20mIU/L)	1.515	0.322	22.083	1	0	4.551	2.419	8.563
	Abnormal (>4.20mIU/L)								
	GCI								
	GCI = 5	−1.468	0.472	9.675	1	0.002	0.23	0.091	0.581
	GCI = 6	0.215	0.287	0.558	1	0.455	1.24	0.706	2.177
	GCI = 7								
	Attempted Suicide								
	No	0.469	0.275	2.909	1	0.088	1.598	0.932	2.738
	Yes								
Overweight	Age (years)	0.293	0.206	2.016	1	0.156	1.341	0.894	2.009
	Age of onset (years)	−0.254	0.208	1.492	1	0.222	0.776	0.517	1.166
	HAMD	0.001	0.029	0.001	1	0.98	1.001	0.945	1.06
	ATPO	−0.001	0	1.806	1	0.179	0.999	0.999	1
	TC	−0.129	0.075	2.943	1	0.086	0.879	0.759	1.019
	TG	0.125	0.066	3.587	1	0.058	1.134	0.996	1.291
	Sex								
	Male	−0.068	0.127	0.285	1	0.593	0.935	0.729	1.198
	Female								
	TSH								
	Normal (0.2–4.20mIU/L)	0.827	0.149	30.835	1	0	2.286	1.707	3.06
	Abnormal (>4.20mIU/L)								
	GCI								
	GCI = 5	0.14	0.207	0.453	1	0.501	1.15	0.766	1.727
	GCI = 6	0.047	0.179	0.068	1	0.794	1.048	0.738	1.489
	GCI = 7								
	Attempted Suicide								
	No	−0.257	0.176	2.13	1	0.144	0.773	0.548	1.092
	Yes								

## 4. Discussion

To our knowledge, this is the first clinical study showing that the prevalence of overweight was 52.89%, and the prevalence of obesity was 4.14% in young Chinese patients with FEDN MDD. Age, age of onset and gender were associated clinical factors for obesity, and TSH was a common risk factor for obesity and overweight in young FEDN MDD patients. In addition, the prevalence of obesity in young MDD patients in this study was higher than in our previous study of MDD patients of all ages (3.73%), while the prevalence of overweight was lower (56.00%) (17), suggesting that younger MDD patients may have a higher risk of obesity than older MDD patients.

Our study found that age was positively correlated with BMI in younger MDD patients, and that the older the patient, the greater the risk of higher BMI. A large US population-based survey on the relationship between risk factors for depression and age showed a stronger association between higher BMI and depression at younger age (24), which is not inconsistent with our present study because the criteria for age grouping were different. Our current study focused on young MDD patients aged 18–35 years, whereas previous US study categorized age into three groups aged 18–39, 40–59, and 60 years, with age being a continuous variable for each subgroup in each 10-year model. It is worthy noting that differences in ethnicity or dietary patterns, as well as the use of antidepressant medication, may influence the relationship between age and BMI to a greater or lesser extent. In the United States, the prevalence of obesity among young adults in 2007–2009 was 29.9% (25), whereas the prevalence of obesity among young Chinese was only 5.6% in 2002 (26). Another survey of young Chinese college students showed that the prevalence of obesity among college students was only 0.4% (27). This study was conducted on young Chinese adults and may differ from the U.S. study in terms of ethnicity and dietary patterns. A previous study also showed that antidepressant use affected patients’ BMI (19).

Further, our study found that age of onset was an associated clinical factor for obesity in young MDD patients. One study found that early-onset depression was associated with a history of depressive episodes and a longer duration of symptoms (28). Another study also showed that patients with early-onset depression had higher levels of neuroticism and a more prevalent abnormal personality (29). Therefore, we hypothesized that a long history of depressive episodes, symptom duration, and higher levels of neuroticism would correlate with patients’ appetite/BMI, making the age of onset an associated clinical factor for obesity in young MDD patients.

In addition, we found significant gender differences in the risk of obesity in young MDD patients. Specifically, young male MDD patients had a higher risk of obesity than young female MDD patients. This interesting finding is inconsistent with previous studies. A study based on US patients showed that female MDD patients had a substantially higher BMI than male patients (30). Another survey of young Americans showed that obese women were more likely to suffer from depression than non-obese women, but there was no significant difference among young male patients (2). This opposite gender difference between young Chinese and young American adults may be due to different criteria for age grouping and dietary patterns, which needs to be explored in future studies.

HAMD score reflects the severity of depression and several surveys have shown that BMI is positively correlated with depression severity in patients with MDD (14, 31), which is consistent with our current study. Possible reasons for this discrepancy may be due to differences in ethnicity, heterogeneity in medication use, disease duration, and inclusion criteria. Moreover, our previous study also showed that HAMD score was a risk factor for weight gain only within two specific score ranges (28 < HAMD score < =29 and > 32) (17). Therefore, more large-sample surveys and animal studies are needed to confirm the relationship between HAMD score and BMI.

TSH is a unique associated clinical factor for overweight and obesity in young MDD patients, which is consistent with our previous findings in all-age FEDN MDD patients (17). This phenomenon can be explained by biological factors, such as a decrease in 5-hydroxytryptamine, an important neurotransmitter in the formation of MDD, which leads to increased concentrations of thyrotropin-releasing hormone and consequently to thyrotropin secretion in brain tissue (32). Another study also found that elevated cortisol concentrations were strongly associated with the development of MDD disease (33). Patients with depression tend to over-activate the HPA axis, the end product of which is cortisol, and elevated cortisol levels decrease the activity of 5-HT1A receptors in the brain, which in turn exacerbates depression (34). It is also reasonable to find no independent association of either FT3 or FT4 with both obesity and overweight in young MDD patients. Although TSH, FT3, and FT4 are critical criteria for thyroid function, changes in TSH do not always correlate positively with changes in FT3 or FT4. For example, subclinical hypothyroidism is defined by elevated TSH (> 4.2 mIU/L) and normal FT4 (35), whereas hyperthyroidism is recognized by low TSH, high FT4, or high FT3 (14). Further animal studies are needed to reveal the relationship between TSH, FT3, FT4, and BMI.

We found that CGI was an associated clinical factor for obesity in young MDD patients. In the group of obese subjects, CGI of 5 appeared to be much less frequent, suggesting that obese MDD patients have more severe disease. Similarly, a previous study also found that higher levels of CGI were a risk factor for obesity in pediatric and adolescent MDD patients (36). All of these findings suggest that close monitoring of weight changes in clinical care is important for young MDD patients.

This study still has several limitations. First, the current international definition of youth is inconsistent. For the age definition of youth, we used an age range that is more appropriate for the Chinese population, which may lead to inconsistent results with other studies. Second, we used BMI rather than waist/hip circumference (WC) to measure obesity/overweight. Although BMI is a widely accepted screening measure for overweight and obesity, other studies have found that waist circumference rather than BMI explains the health risks associated with obesity (37). Therefore, it may be better to calculate both BMI and WC. Third, our participants included only FEDN MDD outpatients in the Chinese Han population, and our findings may not be generalized to MDD patients in other settings. Fourth, previous studies have shown that low-weight MDD patients are of significant research value. However, only two of the 917 young FEDN MDD patients in this study were underweight. In future studies, we will expand the sample size and investigate the prevalence of low weight and associated risk factors in MDD patients.

## 5. Conclusion

In summary, our study showed for the first time that the prevalence of overweight and obesity among young FEDN MDD patients was 52.89 and 4.14%, respectively. Our study also found that TSH was a common risk factor for obesity and overweight in young MDD patients. Therefore, TSH testing in MDD patients should be enhanced in clinical practice, which may be predictive of BMI changes in younger patients. In addition, key population information (age, age of onset, and gender) can be screened in clinical practice to guide patients on weight control, which is important to improve the prognosis of MDD.

## Data availability statement

The data that support the findings of this study are available from the corresponding author upon reasonable request.

## Ethics statement

The studies involving humans were approved by Institutional Review Board (IRB) of the First Hospital of Shanxi Medical University. The studies were conducted in accordance with the local legislation and institutional requirements. The participants provided their written informed consent to participate in this study.

## Author contributions

J-JZ: Conceptualization, Formal analysis, Funding acquisition, Investigation, Supervision, Writing – original draft, Writing – review & editing. X-QW: Data curation, Formal analysis, Writing – original draft, Writing – review & editing. QZ: Writing – review & editing. NG: Writing – review & editing. X-YZ: Conceptualization, Funding acquisition, Resources, Supervision, Writing – review & editing.
